# Multimodality Imaging of Progressive Aortic Valve Endocarditis: From Endophthalmitis to Aorto-Atrial Fistula

**DOI:** 10.7759/cureus.31239

**Published:** 2022-11-08

**Authors:** Aditya Achanta, Yaman Kherallah, Emily Hartsough, Madeleine I Matthiesen, Albree Tower-Rader, Emily K Zern

**Affiliations:** 1 Department of Medicine, Massachusetts General Hospital, Boston, USA; 2 Department of Anesthesia, Critical Care, and Pain Medicine, Massachusetts General Hospital, Boston, USA; 3 Department of Pathology, Massachusetts General Hospital, Boston, USA; 4 Department of Cardiology, Corrigan Minehan Heart Center, Massachusetts General Hospital, Boston, USA

**Keywords:** valvular heart disease, heart failure, echocardiography, acute endophthalmitis, coronary computed tomography angiogram (ccta), aortic endocarditis, aorto-atrial fistula

## Abstract

Our case highlights an atypical presentation of aortic valve endocarditis after initial presentation with endophthalmitis. This case demonstrates the rapidity of evolution of aortic valve endocarditis through sequential, multimodal imaging, and features the importance of a multidisciplinary approach required for the management of complicated aortic valve endocarditis. A male in his mid-thirties was admitted to the hospital with left endophthalmitis and diabetic ketoacidosis. He was found to have aortic valve endocarditis and severe aortic insufficiency, which progressed to aortic root pseudoaneurysm and subsequently to aorto-atrial fistula in less than 72 hours, as demonstrated by consecutive multimodality imaging studies. After extensive surgical repair, post-operative recovery, and rehabilitation, he was discharged home with a good functional outcome. Sequential and multimodal imaging can be beneficial in diagnosing paravalvular infection early in its evolution, which is crucial for decision-making regarding medical and surgical treatment strategies.

## Introduction

Aorto-cavitary fistula formation is an uncommon complication of endocarditis and is estimated to occur in less than 1% of cases to up to 1.6% of cases [1,2]. Due to the complexity of valvular and paravalvular pathology in endocarditis and the inherent limitations of each cardiac imaging strategy, multimodal imaging is often required to elucidate the extent of infection [3,4].

We report a case of a man in his mid-thirties with previously undiagnosed diabetes mellitus who presented with endogenous endophthalmitis and diabetic ketoacidosis as the initial presentations of pneumococcal aortic valve endocarditis. Multimodal imaging demonstrated the sequential and rapid progression from diagnosis to formation of aorto-atrial fistula.

## Case presentation

A man in his mid-thirties presented to our institution with left endophthalmitis. On presentation, he was diagnosed with diabetic ketoacidosis after he was found to have a blood glucose level greater than 400, ketosis, anion gap metabolic acidosis, and hemoglobin A1c of 11%. Despite the absence of pulmonary symptoms, his screening nasopharyngeal coronavirus disease 19 (COVID-19) PCR test returned positive. His blood cultures initially grew high-grade *Streptococcus pneumoniae*; with subsequent culture maturation, *Staphylococcus epidermidis* and *Staphylococcus lugdunensis* were also identified. After day 1 of admission, his cultures cleared and no further organisms were identified.

**Figure 1:**
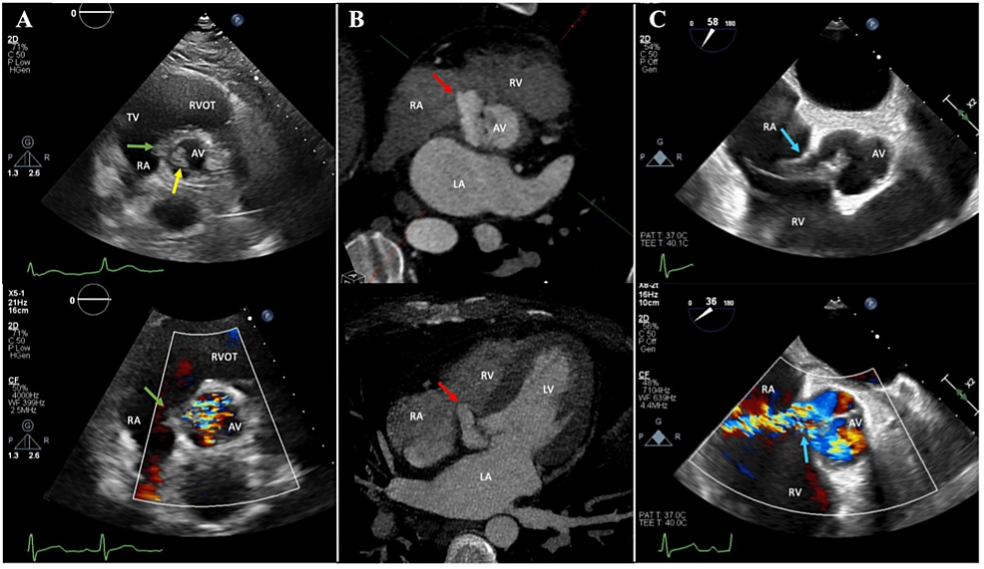
Rapid progression of aortic valve endocarditis. (A) Transthoracic echocardiographic imaging (day 3) shows aortic valve endocarditis (yellow arrow) complicated by severe aortic insufficiency with concern for extension into the aortic root (green arrows). (B) Cardiac computed tomography (day 4) demonstrates pseudoaneurysm of the noncoronary cusp of the aortic valve (red arrows). (C) Transesophageal echocardiogram (day 5) shows rupture of the aortic root pseudoaneurysm with fistula formation between the aortic root and right atrium as demonstrated by color Doppler (blue arrows). Abbreviations: AV, aortic valve; LA, left atrium; LV, left ventricle; RA, right atrium; RV, right ventricle; RVOT, RV outflow tract; TV, tricuspid valve

On day 2 after admission, a new loud diastolic murmur in the left upper sternal border was noted. On day 3 after admission, transthoracic echocardiography was notable for an aortic valve echodensity on the noncoronary cusp, moderate-to-severe aortic regurgitation, and an echodensity between the aortic root and base of the septal leaflet of the tricuspid valve measuring 6.5 x 8 mm without flow connection by color Doppler, concerning for early extension of endocarditis (Figure 1A). A gated cardiac computed tomography angiography (cardiac CTA) obtained on day 4 demonstrated a larger pseudoaneurysm of the noncoronary cusp measuring 14 x 10 x 20 mm projecting into the tricuspid annulus without fistulous communication; the aortic valve noncoronary cusp thickening and a tricuspid valve hypodensity consistent with endocarditis were redemonstrated (Figure 1B). Also on day 4, CT angiography of the head showed no evidence of embolic events affecting the brain parenchyma. On day 5, a transesophageal echocardiogram (TEE) demonstrated a new fistula between the aortic root (in the regions of the noncoronary and right coronary cusps) and the right atrium (Figure 1C). Severe aortic regurgitation and multiple vegetations of the aortic and tricuspid valves were also visualized.

Determination of the optimal treatment approach strategy was challenging due to multiple sources of infection competing with the ultimate need for cardiac surgery. Multidisciplinary discussions were held between cardiac surgery, cardiology, internal medicine, ophthalmology, and infectious disease specialists. In light of his relative clinical stability with only mild heart failure signs and symptoms, no clinical pulmonary sequalae of COVID co-infection, and no high-grade conduction disease, the patient underwent pre-operative optimization with left eye enucleation, dental extractions, and a full week of antibiotic therapy.

**Figure 2:**
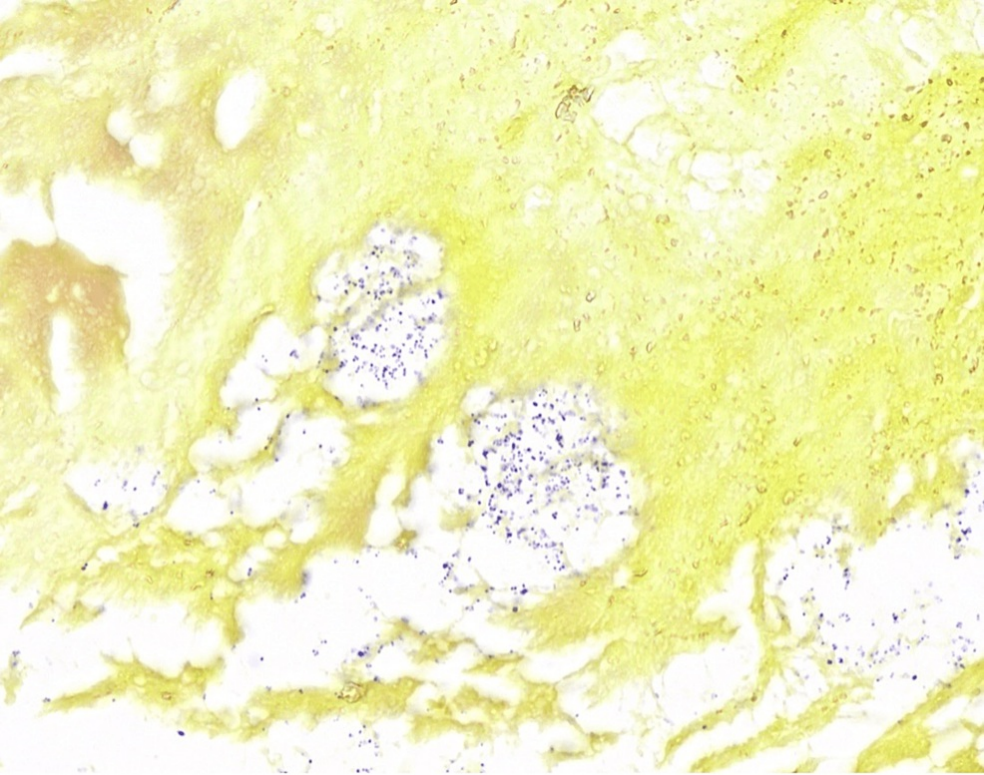
Tissue gram stain (1000X) showing small gram-positive cocci predominantly of one morphotype consistent with the culture finding of Streptococcus pneumoniae. A second morphotype corresponding to the culture finding of two organisms was not definitive.

On day 8, the patient underwent aortic root replacement, aortic valve replacement with a 25-mm bioprosthetic valve, repair of the aorta-to-right atrial fistula, removal of a vegetation encroaching on the base of the septal leaflet of the tricuspid valve, and placement of a pericardial patch to the right atrial wall. Diagnostic pathology of the aortic valve and aortic root surgical specimens demonstrated gram-positive cocci (Figure 2).

**Figure 3:**
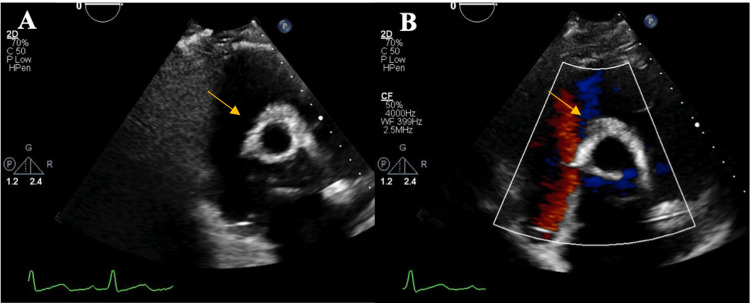
Post-operative transthoracic echocardiogram demonstrating bioprosthetic aortic valve. (A) After the patient underwent replacement of the aortic valve and root replacement and repair of the right atrial wall, follow-up transthoracic echocardiography demonstrated a well-seated bioprosthetic aortic valve (yellow arrow) without valvular or paravalvular regurgitation. (B) Color Doppler showing no persistent aorto-atrial fistula.

His post-operative course was complicated by complete heart block requiring a dual-chamber permanent pacemaker, a polymorphic ventricular tachycardia arrest (mediated by long QT interval and bradycardia), and pericardial effusion necessitating drainage. On day 32, transthoracic echocardiogram demonstrated normal biventricular systolic function, a well-functioning bioprosthetic valve, and trace tricuspid regurgitation (Figure 3). He was discharged to an acute care rehabilitation facility with 2.5 weeks of intravenous antibiotic therapy with outpatient follow-up with cardiac surgery, cardiology, infectious disease, and ophthalmology services. After rehabilitation, he was discharged home with a good functional outcome.

## Discussion

Aorto-cavitary fistula is an uncommon complication of native valve infectious endocarditis, and this case uniquely highlights the three-day evolution from detection of cardiac murmur and diagnosis to development of aorto-atrial fistula demonstrated by multimodality advanced cardiac imaging. Despite appropriate antibiotic therapy at time of presentation, progressive valvular and paravalvular aortic infection occurred in our patient, as demonstrated on daily sequential echocardiographic and cardiac CTA. Clinicians must maintain a high index of suspicion for progressive paravalvular infection, even in the context of blood culture negativity and minimal change in clinical status, as ongoing infection and inflammation are possible at the tissue level. Repetitive imaging with multimodal imaging strategies can help inform medical treatment or urgency of surgical intervention. In our case, multidisciplinary care and pre-operative optimization of infectious comorbidities and their predisposing factor (severe diabetes mellitus) allowed for successful surgical and medical treatment of complicated aortic valve and aortic pathology.

In a systematic review of case reports conducted in 2019, endocarditis was noted as the most common cause of aorto-atrial fistula and was responsible for 23% of cases; in over 70% of cases of endocarditis-related aorto-atrial fistula, paravalvular abscess was also identified [5]. Aorto-cavitary fistula formation occurred in approximately 1.6% of infective endocarditis cases [2]. In a review of 76 cases of aorto-cavitary fistula formation, *Staphylococcus aureus*, *Staphylococcus epidermidis*, and *Streptococci* were the three most commonly identified organisms to cause aorto-cavitary fistula [2]. Transthoracic echocardiography detected fistula in 53% of cases, which improved to 97% of cases with TEE [2]. The median duration of time from onset of symptoms to echocardiographic diagnosis of endocarditis and aorto-cavitary fistula has been reported to be 19 days and 25 days, respectively, and the median time from diagnosis of endocarditis to diagnosis of fistula was 5 days [2].

Guidelines for the management of patients with valvular heart disease from the American College of Cardiology/American Heart Association (2020) and for the management of infective endocarditis from the European Society of Cardiology (2015) comment on the nuanced role of multimodality imaging in the diagnosis of endocarditis, monitoring progression, evaluating for endocarditis related complications, and pre-operative evaluations [6,7]. Echocardiography and cardiac CTA are widely used imaging modalities to detect native valve endocarditis and assess for complications, though there are clinical and diagnostic limitations to both modalities. While in some patients, clinicians may have heightened concern for the iodinated contrast utilization and radiation exposure of cardiac CTA, TEE carries a risk of potential procedural complications and requires procedural planning. In our patient, logistical considerations presented an initial barrier to TEE scheduling due to special airborne precautions in light of his COVID-19 infection. Cardiac CTA may fail to identify small isolated vegetations, and echocardiographic techniques may have limited ability to detect paravalvular infection, particularly if there are limitations to imaging quality, such as calcification or prosthetic material. Therefore, synergy between these modalities, as demonstrated in our patient case, may be required to comprehensively diagnose the extent of endocarditis and determine treatment plan. Though not utilized in our patient with native valve endocarditis, nuclear molecular techniques are evolving as a supplementary method of endocarditis assessment in patients with diagnostic difficulty and/or suspected endocarditis in the presence of prosthetic valve or implantable electronic device [4,7].

## Conclusions

Overall, our case highlights an atypical presentation of aortic valve endocarditis after an initial presentation of endophthalmitis. Furthermore, this case demonstrates the rapidity of evolution of aortic valve endocarditis through multimodal imaging, and features the importance of a multidisciplinary approach required for the diagnosis and management of complicated aortic valve endocarditis. Sequential and multimodal imaging can be beneficial in diagnosing paravalvular infection early in its evolution, which is crucial for decision-making regarding medical and surgical treatment strategies.
